# RelB activation in anti-inflammatory decidual endothelial cells: a master plan to avoid pregnancy failure?

**DOI:** 10.1038/srep14847

**Published:** 2015-10-14

**Authors:** Elisa Masat, Chiara Gasparini, Chiara Agostinis, Fleur Bossi, Oriano Radillo, Francesco De Seta, Nicola Tamassia, Marco A. Cassatella, Roberta Bulla

**Affiliations:** 1 Department of Life Sciences, University of Trieste, Trieste, Italy; 2 Institute for Maternal and Child Health, IRCCS (Istituto di Ricovero e Cura a Carattere Scientifico) “Burlo Garofolo”, Trieste, Italy; 3 Section of General Pathology, Department of Medicine, School of Medicine, University of Verona, Verona, Italy

## Abstract

It is known that excessive inflammation at fetal-maternal interface is a key contributor in a compromised pregnancy. Female genital tract is constantly in contact with microorganisms and several strategies must be adopted to avoid pregnancy failure. Decidual endothelial cells (DECs) lining decidual microvascular vessels are the first cells that interact with pro-inflammatory stimuli released into the environment by microorganisms derived from gestational tissues or systemic circulation. Here, we show that DECs are hypo-responsive to LPS stimulation in terms of IL-6, CXCL8 and CCL2 production. Our results demonstrate that DECs express low levels of TLR4 and are characterized by a strong constitutive activation of the non-canonical NF-κB pathway and a low responsiveness of the canonical pathway to LPS. In conclusion, DECs show a unique hypo-responsive phenotype to the pro-inflammatory stimulus LPS in order to control the inflammatory response at feto-maternal interface.

Human endothelial cells (ECs) are multifunctional cells that line blood vessels, capable to secrete a variety of biologically active mediators. In disease states, ECs are activated by several mechanisms enhancing the expression of cell adhesion molecules and cytokines, in order to orchestrate the inflammatory response^1^. ECs, although similar in function and morphology, represent an heterogeneous population of cells in terms of secretion of inflammatory mediators, modulation of adhesion molecules, leakiness and pro-coagulant activity^2^. LPS is a strong activator of ECs^3^, and is recognized by a multi-receptor complex composed by TLR4 and myeloid differentiation-2 (MD2)^4^ through the involvement of LPS-binding protein (LBP) and CD14. TLR4 transduces its signal through two main pathways: the MyD88-dependent pathway, which mediates the early NF-κB activation, and the MyD88-independent pathway (TRIF-dependent pathway), which mediates the delayed NF-κB activation^5^.

The NF-κB family is composed of 5 subunits, p65, p50, RelB, p52 and c-rel, that act as homo- and hetero-dimers, with the exception of RelB, which is found in its active form only as RelB/p50 or RelB/p52 hetero-dimers. The canonical NF-κB pathway is mediated by the dimer p65/p50, has an early activation kinetic and is involved in pro-inflammatory responses, cell survival and innate immunity. A second pathway, that relies on RelB/p50 and RelB/p52, has been characterized with a delayed activation kinetic^6^. It modulates the secondary lymphoid organ development, acquired immunity^7^ and, very interestingly, downregulates inflammation^8^.

Excessive inflammation at fetal-maternal interface is thought to be a key contributor in a compromised pregnancy. Intrauterine infections have been associated with pregnancy complications such as preterm labor, intrauterine growth restriction and pre-eclampsia^9^. Due to their location and properties, the microvascular ECs that line decidual vessels are very likely to play an important role in the control of the inflammatory response at feto-maternal interface.

In this study we investigated how decidual endothelial cells respond to LPS in terms of cytokine production, expression of TLR4 and signal transduction.

## Results and Discussion

### Production of pro-inflammatory cytokines by endothelial cells

**Figure 1:**
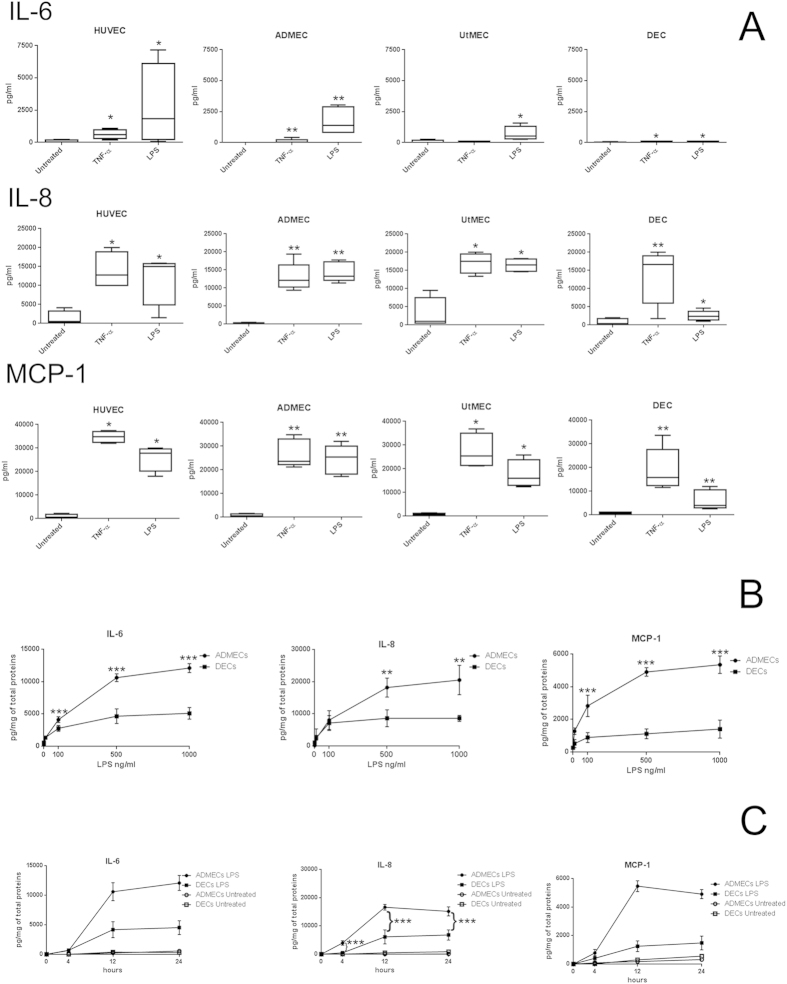
Cytokine and chemokine production by ECs after stimulation with inflammatory stimuli. (**A**) The box plots represent the production of IL-6, CXCL8 (IL-8) and CCL2 (MCP-1) in the supernatants of confluent monolayers of HUVECs, ADMECs, UtMEC and DECs. All the ECs examined produced high level of cytokines and chemokines in response to TNF-α and LPS excepted DECs which produced low levels of these pro-inflammatory cytokines. Box plot ± S.D. of at least 5 independent experiments. *P < 0.05 and **P < 0.01 untreated versus treated (Mann-Whitney test). (**B**) Dose response curve of LPS effect on DECs and ADMECs in the cytokines production. The cells were stimulated with increasing concentrations of LPS (0 ng/ml, 10 ng/ml, 100 ng/ml, 500 ng/ml and 1000 ng/ml) and the cells supernatants were analyzed for the presence of of IL-6, CXCL8 (IL-8) and CCL2 (MCP-1). If compared to ADMECs, DECs are poorly respondent to LPS stimulation and at 100 ng/ml of LPS DECs reach the maximum of cellular activation. (**C**) Time course of LPS effect on ECs in the cytokines production. The cells were stimulated for 4 h, 12 h or 24 h with LPS and the cells supernatants were analyzed for cytokine production. The maximum amount of cytokines produced by DECs was at 12 h. Data from three independent experiments are shown and represent the mean ± SD. *P < 0.05 (Mann-Whitney test) ADMECs versus DECs.

We compared the production of the pro-inflammatory cytokines IL-6, CXCL8 (IL-8) and CCL2 (MCP-1) upon stimulation with TNF-α or LPS in DECs, ADMECs, UtMECs and HUVECs. As expected^10^, HUVECs promptly respond to TNF-α or LPS producing IL-6, CXCL8 and CCL2 (Fig. 1). Similar results were obtained in ADMECs, and UtMECs. Interestingly, a very low production of these cytokines was observed when DECs were stimulated by LPS. DECs’ low responsiveness does not seem to be time or dose dependent as assessed by time course and dose-response experiments on DECs and ADMECs (Fig. 1B). We were not surprised about this atypical behaviour of DECs upon LPS challenge, since we previously observed that LPS enhances the expression of inflammasome components and induce IL-1β secretion in trophoblasts and in decidual stromal cells but not in DEC^11^.

### DECs have a lower TLR4 expression and a constitutive activation of the NF-κB non- canonical pathway

**Figure 2:**
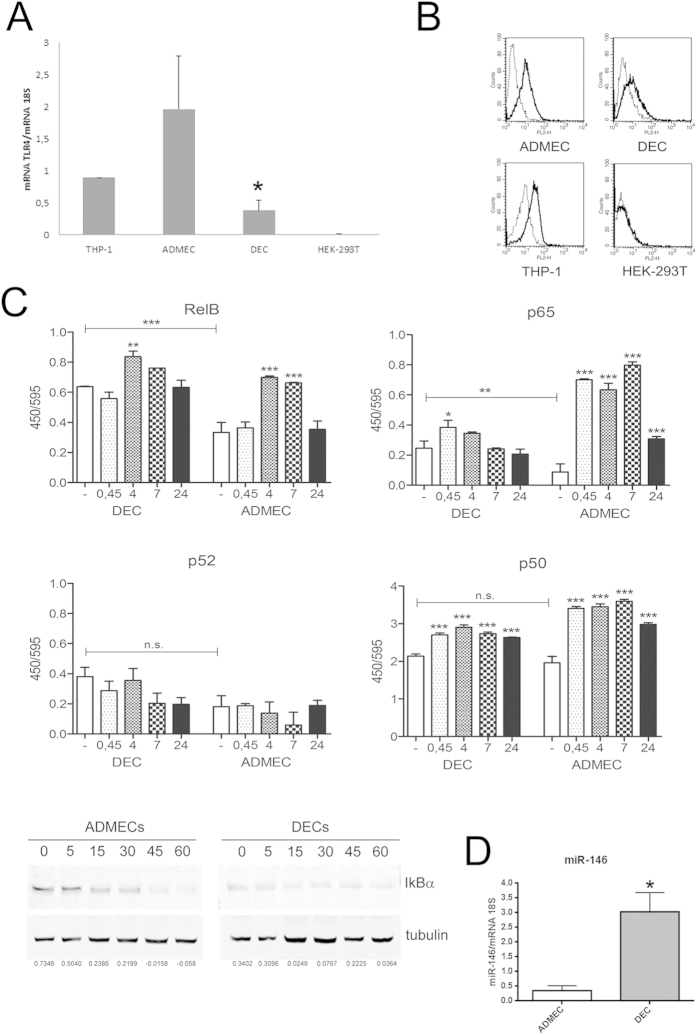
Expression of TLR4, NF-kB pathways and miRNA in ECs. (**A**) Analysis of RT-qPCR for the expression of TLR4 mRNA in EC populations. The relative amount of mRNA for TLR4 in DECs, ADMECs, HEK 293T and THP-1 was evaluated by RT-qPCR and normalized with reference to the 18S value. The results were expressed as AUs, in which 1 AU represents the value obtained with macrophages (PBMC) used as a calibrator. Bars represent the mean ± S.E.M. of at least 3 independent experiments. *P < 0.05 (Mann-Whitney test) ADMECs versus DECs. (**B**) Cytofluorimetric analysis for TLR4 surface expression on freshly isolated ECs (ADMECs and DECs). DECs present a lower expression of TLR4 both at gene and protein level. THP-1 were used as positive control and HEK 293T were used as negative control for TLR4 expression. (**C**) Activation of the signal transduction pathway in ECs. DECs and ADMECs were treated with LPS during a time course of 45 min, 4 h, 7 h and 24 h. Cells were then lysed and nuclear extracts assayed with the TransAm NF-κB family kit (Active Motive) to measure the binding of the subunits RelB, p65, p52 and p50 to their consensus sequence. Data are shown as mean ± SEM of two independent experiments. Statistical analysis were carried out according to Material and Methods. *P < 0.05; **P < 0.01, ***P < 0.001 were calculated versus the unstimulated control of the same cell type, except when the conditions analyzed are clearly connected by a straight line (comparison of the unstimulated conditions between the two different cell types). In the bottom a western blot of DECs and ADMECs lysates is reported to evaluate IkBα degradation, which is indicative of the activation of the NFkB canonical pathway. (**D**) Evaluation of the expression of miR-146 where the relative amount of miR-146 in untreated DECs and ADMECs was evaluated by RT-qPCR. Untreated DECs displayed 6-fold increase the level of miR-146. Bars represent the mean ± S.E.M. of at least 3 independent experiments. *P < 0.05 (Mann-Whitney test) ADMECs versus DECs.

The results in Fig. 2A show that DECs express lower TLR4 mRNA levels than ADMECs. The flow cytometric analysis in Fig. 2B confirmed the RT-qPCR data, showing that TLR4 is present on EC surface, even though its expression on DECs is very weak compared to the high expression on ADMECs. RT-qPCR experiments, summarized in Supplemental Fig. 1, show that both population of ECs, unlike macrophages used as a positive control, do not express the mRNA for CD14; interestingly DECs have also a trend to a reduced expression of the MD2, MyD88 and TRIF mRNA. These data are in agreement with Wang and colleagues who observed a significant downregulation of MyD88 and TRIF in the LPS tolerant glial cells^12^.

Since LPS induces a weak pro-inflammatory response in DECs, we aimed to investigate the activation of the NF-κB pathways, that play a central role in modulating both pro-inflammatory and anti-inflammatory conditions^13^. The activation of the subunits p65 and p50 (canonical pathway) and of the subunits RelB, p50 and p52 (non-canonical pathway) was measured over a time-course (Fig. 2C). The p65 subunit is strongly activated by LPS in ADMECs from 45 min to 24 h. In DECs, p65 shows a certain basal activation and is mildly activated at 45 min stimulation with LPS, but not at later time points (Fig. 2C). It is evident that the canonical pathway has not a major role in DECs functions, as compared to the LPS-responsive cells.

The subunit RelB shows a retarded activation, as expected^6^. Upon LPS stimulation both cell types are characterized by a strong activation of RelB. Very interestingly, RelB shows a high basal activation in DECs which is not observed in ADMECs (Fig. 2C) and, more importantly, such basal activation is not far from its full LPS-dependent activation. In both cell types, p52 is not activated in response to LPS while p50 shows a strong basal activation, that clearly increases after LPS stimulation.

RelB basal activation and p65 low responsiveness to LPS activation seem to be the most relevant features of DECs. A similar basal activation has been observed in cancer^14^ or in endotoxin tolerance. In endotoxin tolerance, RelB has a key role in silencing or inhibiting the expression of the pro-inflammatory cytokines TNF-α and IL-1β^15^ and, when overexpressed, the dimer RelB/p50 inhibits the production of TNF-α in primary dendritic cells and macrophages^8^. Together with RelB activation, unstimulated DECs showed a reduced expression of IκBα (Fig. 2C), the physiological inhibitor of the canonical pathway, that only slightly diminishes after LPS stimulation. This evidence would sustain the observed basal activation of p65 and its low responsiveness to LPS. In ADMECs, IκBα expression is high in unstimulated cells, and rapidly decreases upon LPS activation, as occurring in LPS-responsive cells where the canonical pathway has a major role. Recently, miR-146 has been reported to regulate RelB expression in TNF-α stimulated cells^16^ and to repress endothelial activation^17^. We verified that miR-146 is highly expressed in untreated DECs as compared to ADMECs (Fig. 2D) and therefore could have a role in regulating RelB activity in these cells.

To conclude, DECs are characterized by a strong constitutive activation of the NF-κB non-canonical pathway and a low responsiveness to LPS. Our finding shows a snapshot of a very atypical endothelium that, in order to avoid pregnancy failure, increases the activation threshold to LPS. This is in harmony with the concept of immune tolerance to microorganism during pregnancy^18^.

## Materials and Methods

### Tissues samples

Decidual first trimester biopsy specimens were obtained from women undergoing voluntary termination of pregnancy at 8–12 weeks’ gestation. Skin samples were obtained from women of fertile age undergoing reductive plastic surgery. Endometrial tissue specimens were obtained from fertile women undergoing hysterectomy for leiomyomatosis in the mid proliferative and mid secretory phase defined according to Noyes criteria^19^. The study was approved by the institutional review board of The Maternal-Children’s Hospital (IRCCS “Burlo Garofolo”, Trieste, Italy) and informed consent was obtained from all patients providing the tissue specimens. All the experiments were carried out in accordance with the approved guidelines.

### Cell isolation and culture

Human umbilical vein endothelial cells (HUVECs) were isolated by collagenase treatment and cultured as previously published^20^. Decidual endothelial cells (DECs) and Human dermal microvascular endothelial cells (ADMECs) were isolated and characterized as previously described by Bulla *et al.*^21^.

DECs were positively selected with Dynabeads M-450 (Life Technologies) coated with Ulex europaeus 1 lectin (Sigma–Aldrich), whereas ADMECs were further purified from a subconfluent mixed cell population with the CD31-conjugated magnetic beads from Dynabeads (Life Technologies).

Uterine microvascular endothelial cells (UtMECs) were purified from endometrial tissue samples by a similar procedure for DECs isolation. Briefly, the tissue was finely minced, digested first with 0.25% trypsin (Sigma-Aldrich, Milan, Italy) and 50 μg/ml DNase I (Roche, Milano, Italy) overnight at 4 °C, and then with collagenase type 1 (3 mg/ml) (Worthington Biochemical Corporation, DBA, Milan, Italy) for 30 min at 37 °C. After the filtration through a 100 μm pore nylon filter (BD Falcon, Milan, Italy) the cells were positively selected with Dynabeads M-450 (Dynal, Invitrogen, Milano, Italy) coated with Ulex europaeus 1 lectin (Sigma-Aldrich).

All ECs were seeded on 12,5 cm^2^ flask precoated with 2 μg/cm^2^ fibronectin (Roche, Milan, Italy) and maintained in endothelial serum free basal medium (Life Technologies, Monza, Italy) supplemented with 20 ng/ml bFGF (basic Fibroblast Growth Factor), 10 ng/ml EGF (Epidermal Growth Factor) (Life Technologies) 10% of FCS (Life Technologies) and 10% of human serum (Euroclone, Milan, Italy).

### ELISA

The level of IL-6, CXCL8 (IL-8) and CCL2 (MCP-1) were measured by ELISA using a commercial kit (Human IL-6 Instant ELISA, Bender Medsystem, Milan, Italy; Human IL-8 Instant ELISA, Bender Medsystem, Milan, Italy; Human MCP-1 Instant ELISA, Bender Medsystem, Milan, Italy). Each assay was performed according to the manufacturer instructions.

### RNA extraction, cDNA synthesis and Real-time quantitative PCR (RT-qPCR)

Cells RNA were purified with euroGOLD Total RNA Kit (Euroclone, Milan, Italy) according to the supplier’s instructions. miRNA isolation were carried out with miRNeasy Mini Kit (Qiagen, Milan, Italy) according to the supplier’s instructions. Total RNA extracted was reverse transcripted with iScript cDNA Synthesis Kit (Bio-Rad, Milan, Italy). Real-time quantitative PCR (qPCR) was carried out on a Rotor-Gene 6000 (Corbett, Explera, Ancona, Italy) using iQ SYBR Green Supermix (Thermo Scientific Fynnzymes, Milan, Italy). Table 1 show the primer lists used for qPCR. The relative amount of gene production in each sample was determined by the Comparative Quantification (CQ) method supplied as part of the Rotor Gene 1.7 software (Corbett Research)^22^.

### Cytofluorimetric Analysis

ECs, HEK 293T and THP-1 (5 × 10^5^ cells) were incubated with the (HTA125) monoclonal PE-conjugated anti-human TLR4 antibody (Biolegend, Milan, Italy), with monoclonal FITC-conjugated anti-human MD2 antibody (Hycult Biotech, Milan, Italy), or with unrelated antibody for 1 h at 37 °C. The cells were fixed with 1% paraformaldehyde (Sigma-Aldrich) and analyzed for fluorescence on a FACScalibur instrument (BD Falcon) using CellQuest software.

### Immunoblot

After stimulation, ECs were lysed in lysis buffer (25 mM Tris pH 7.5, NaCl 150 mM, Triton X-100 1%, Sodium Deoxycholate 1%, SDS 0,1%, 2 mM EDTA, 1% (v/v) Nonidet P-40, and 1mM DTT) containing inhibitors of proteases (Sigma-Aldrich), and phosphatases (Sigma-Aldrich), and following a 15-min incubation on ice, cell debris were spun down (12,000 × g, 20 min, 4 °C) and the supernatants were frozen and stored at −80 °C. Small aliquots of the various extracts were routinely processed for protein content determination, by using a protein assay kit (Bio-Rad, Milan,Italy). For Western blot analysis, cell extracts were subjected to immunoblots by described procedures^23^. Detection was carried out with Alexa FluorR 680-conjugated goat anti-rabbit Abs (Invitrogen) and IRDye™ 800-conjugated goat anti-mouse IgG (Rockland, Gilbertsville, PA, USA) secondary Abs. Blotted proteins were detected and quantified using the Odyssey infrared imaging system (LI-COR Biosciences, Lincoln, NE, USA). Anti-IκBa (sc-371) was obtained from Santa Cruz Biotechnology while anti-β-tubulin (T5293) was from Sigma-Aldrich.

### DNA binding

DNA binding assay was performed as previously shown^24^. p65, RelB, p52, p50, binding to consensus sequence was assayed in nuclear lysates using the TransAm NF-κB family kit (Active Motif), according to the manufacturer’s instructions. The anti-NF-κB family primary antibodies recognize an epitope that is accessible only when NF-κB is activated and bound to its target DNA.

### Statistical Analysis

Mean, standard deviation (SD), standard error of the mean (SEM) and statistical tests were calculated using GraphPad Prism (GraphPad Software, San Diego, CA). For the ELISA experiments, the Mann Whitney test was used to compare 2 groups of data, P value of <0.05 was considered significant (*p < 0.05 **p < 0.001).

For the DNA binding experiments, statistical analysis was carried out using a 1-way analysis of variance (ANOVA) with Dunnett’s multiple comparison post-test (*p < 0.05; **p < 0.01, ***p < 0.001).

## Additional Information

**How to cite this article**: Masat, E. *et al.* RelB activation in anti-inflammatory decidual endothelial cells: a master plan to avoid pregnancy failure? *Sci. Rep.* **5**, 14847; doi: 10.1038/srep14847 (2015).

## Supplementary Material

Supplementary Information

## Figures and Tables

**Table 1:** Primer used for RT-qPCR analysis.

Sample	Primers	Sequence 5′ > 3′	AnnealingTemperature (°C)	AmpliconSize (bp)	Gene BankAccession Number
18S	Forward	ATCCCTGAAAAGTTCCAGCA	60	154	NM 022551
	Reverse	CCCTCTTGGTGAGGTCAATG			
TLR4	Forward	AAGCCGAAAGGTGATTGTTG	60	153	NM_138557.1
	Reverse	CTGAGCAGGGTCTTCTCCAC			
MD2	Forward	TTCCACCCTGTTTTCTTCCATA	60	404	AB446498.1
	Reverse	GGCTCCCAGAAATAGCTTCAAC			
CD14	Forward	AGAGGCAGCCGAAGAGTTCAC	60	132	NM_000591.3
	Reverse	GCGCTCCATGGTCGATAAGT			
MyD88	Forward	CTCTGTTCTTGAACGTGCGGA	60	246	NM_002468
	Reverse	ACTTTTGGCAATCCTCCTCAATG			
TRIF	Forward	TGCACTGCGTTCTCATAGTCT	61	167	U49441
	Reverse	ACTGTGCCATAGGGTCTGATG			
hsa miR-146	Forward	CGGCTGAATTGGAAATGATA	60	22	MI0000477
	Reverse	TGCTGCCTCTCAAACAGAAG			
