# Supplementary material for: RelB activation in anti-inflammatory decidual endothelial cells: a master plan to avoid pregnancy failure?
Source: Sci Rep. 2015 Oct 14;5:14847. doi: 10.1038/srep14847 (PMC4604455; doi:10.1038/srep14847)
Supplement: Supplementary Information [file srep14847-s1.doc]

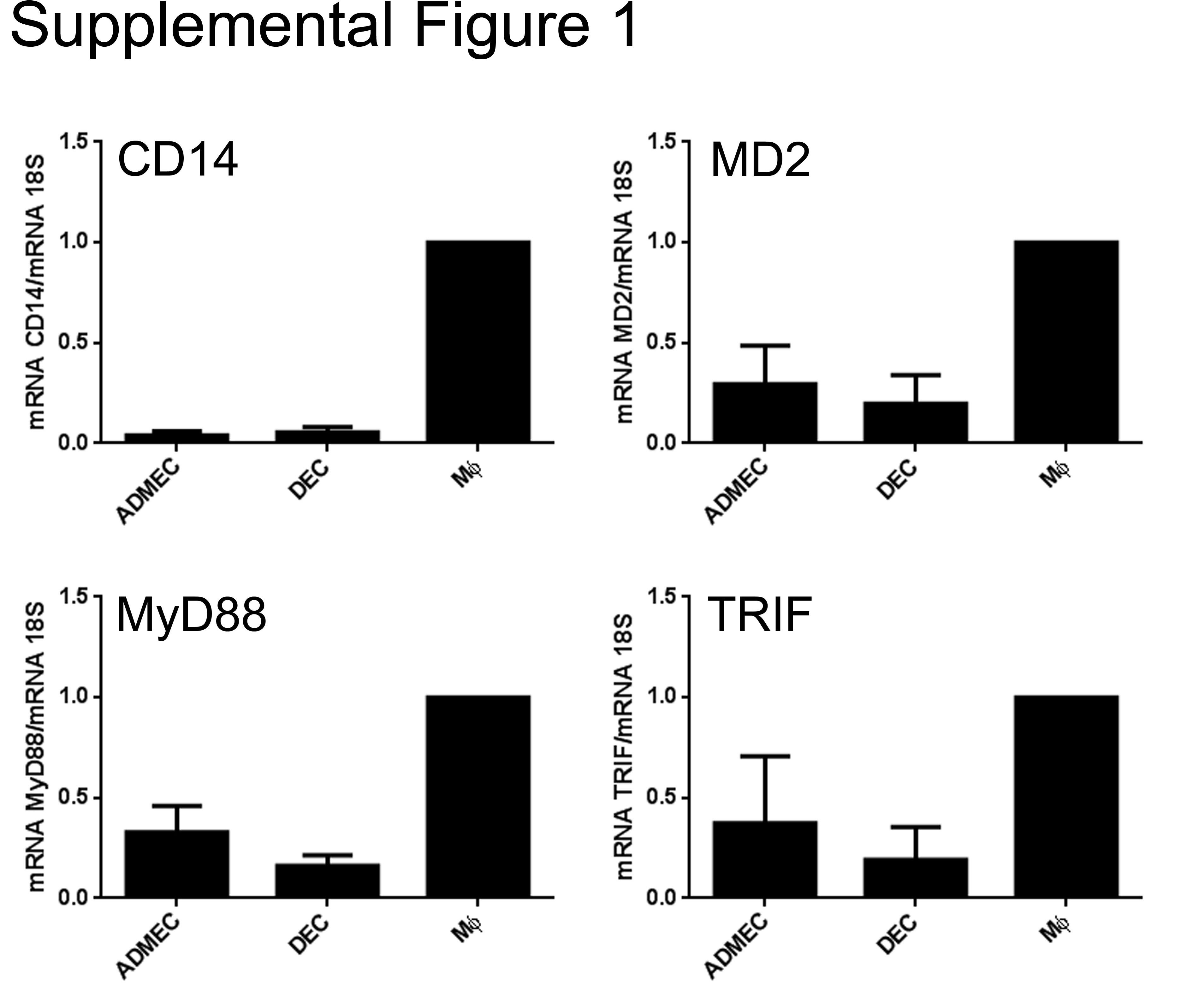


**Supplemental Figure 1. Analysis of RT-qPCR for the expression of CD14, MD2, MyD88 and TRIF mRNA in EC populations.** The relative amount of mRNA for CD14, MD2, MyD88 and TRIF in DECs, ADMECs was evaluated by RT-qPCR and normalized with reference to the 18S value. The results were expressed as AUs, in which 1 AU represents the value obtained with macrophages (PBMC) used as a calibrator. Bars represent the mean ± S.E.M. of at least 3 independent experiments. *P < 0.05 (Mann-Whitney test) ADMECs versus DECs.
